# Synovial folds of the lateral atlantoaxial joints in dogs: a magnetic resonance, anatomic, and histologic study

**DOI:** 10.3389/fvets.2025.1646781

**Published:** 2025-12-04

**Authors:** J. B. Burg-Personnaz, S. Kaessmeyer, F. Forterre, C. Precht, K. Haenssgen

**Affiliations:** 1 Division of Clinical Radiology, Department of Clinical Veterinary Science, Vetsuisse Faculty, University of Bern, Bern, Switzerland; 2 Division of Veterinary Anatomy, Department of Clinical Research and Veterinary Public Health, Vetsuisse Faculty, University of Bern, Bern, Switzerland; 3 Division of Small Animal Surgery, Department of Clinical Veterinary Science, Vetsuisse Faculty, University of Bern, Bern, Switzerland

**Keywords:** meniscoid folds, dog, MRI, cervical, neck

## Abstract

**Purpose:**

Synovial folds of the lateral atlantoaxial joints are well described normal anatomical structures in humans, which play a potential role in neck pain; however, scant information exists regarding these structures in dogs. The purpose of this study was to identify and describe atlantoaxial synovial folds in dogs using magnetic resonance imaging (MRI), gross anatomy and histological examination.

**Methods:**

Six client-owned dogs, euthanized for reasons unrelated to this study and without known disease in the craniocervical region, underwent postmortem MRI of the cranial cervical spine (3 Tesla, Siemens Magnetom Vida) using T2-weighted (T2w), T1-weighted (T1w), short tau inversion recovery (STIR), Dixon, and fat-suppressed volumetric interpolated breath-hold examination (VIBE) sequences. In five dogs the lateral atlantoaxial joints were gross anatomically dissected, while one of them underwent histological examination.

**Results:**

Fat-suppressed VIBE sequences provided optimal visualization of synovial folds within the atlantoaxial joints. Ventral synovial folds were discernible in all dogs by MRI and gross anatomical examination presenting as ventral synovial bulges extending into the joint space. While smaller dorsal synovial folds were identified by MRI only in 2 dogs, but by gross anatomical examination in all dogs. Light microscopy of the histological specimen confirmed that the folds were extensions of the joint capsule’s synovial membrane, which were mainly composed of vascularized connective and adipose tissue; and were covered by synovial cells.

**Discussion/conclusion:**

Synovial folds are present in canine atlantoaxial joints free from known atlantoaxial disease. Dorsal and ventral folds could be visualized on post-mortem macroscopic and histological examinations. The larger ventral folds were consistently visualized using MRI with fat-suppressed VIBE sequences. The anatomical structure of the canine synovial folds suggests a similarity to humans, indicating that some conditions may analogously affect the synovial folds. Further investigations are warranted to elucidate the clinical relevance of these synovial folds in dogs.

## 1 Introduction

The atlantoaxial synovial folds have been described in detail as physiological anatomical structures in human medicine, providing insights into their morphology, function, and potential involvement in clinically relevant disorders (1–3). In humans, the atlantoaxial joint is composed of three distinct joint cavities, the median, left and right atlantoaxial joint cavities, which sometimes intercommunicate. Additionally, the *Bursa atlantodentalis,* located between the *Dens axis* and the *Ligamentum transversum atlantis* sometimes communicates with the median atlantoaxial joint cavity (4).

The atlantoaxial and atlantooccipital joints in dogs form one large synovial cavity, which is subdivided into a pair of lateral atlantooccipital cavities, a median cavity that runs between the dens and the atlas, and in addition the paired left and right atlantoaxial joint cavities (5). This means, that in dogs, the atlantoaxial joint cavities intercommunicate and communicate with the atlantooccipital joint cavities, and this intercommunication sometimes also occurs in humans (5).

**Figure 1:**
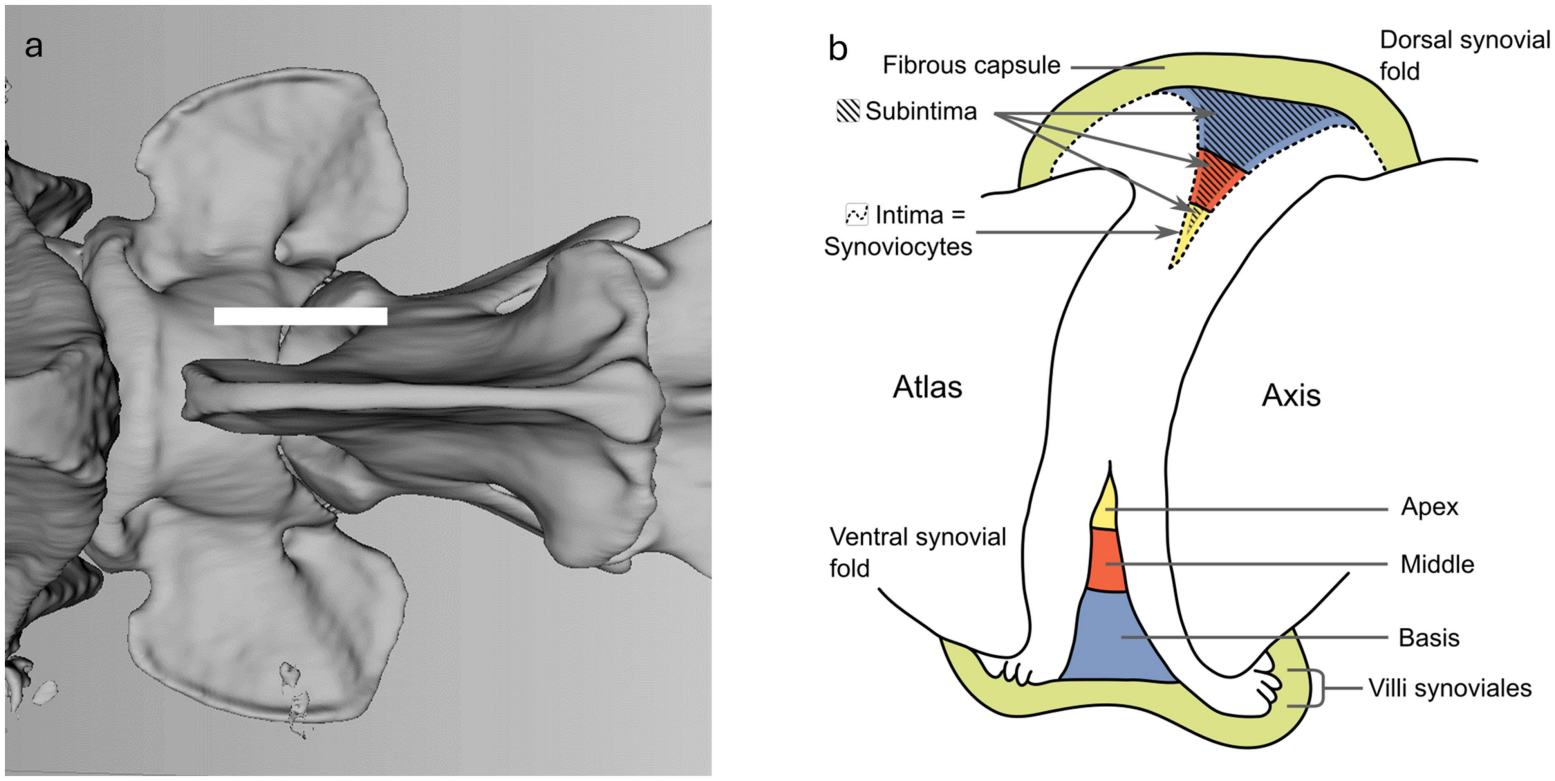
**(a)** Dorsal 3D volume rendering view of the atlantoaxial joint. The white line shows the location of the parasagittal section of the schematic visualization in **b**. **(b)** Schematic visualization of a parasagittal section through the atlantoaxial joint, demonstrating the outer fibrous layer and the inner synovial layer. The subdivision of the synovial fold is demonstrated in blue, red and yellow. Adapted from Webb et al. (3).

In general the joint capsule consists of an outer fibrous layer (*Stratum fibrosum*), and an inner synovial layer (*Stratum synoviale*), the latter can be further subdivided in intima and subintima (Figure 1) (4). The synovial layer forms physiological protrusions or synovial folds at various points of the joint capsule, some of which protrude into the intraarticular space (Figure 1). The intima consists of epithelial-like cells, the synoviocytes and the subintima, also referred to as subintimal stroma which fills the space between the synoviocytes and the outer fibrous layer. The synoviocytes form a discontinuous cell layer, and lack a basement membrane (4). Two types of synoviocytes can be distinguished; macrophage-like cells and fibroblast-like cells, the existence of both cell types was confirmed in humans and dogs amongst other species (6). The synoviocytes are arranged in layers of one to three cells. A differentiation between both types of synoviocytes is limited using light microscopy and requires immunohistochemistry and/or transmission electron microscopy (6). The subintima merges with the outer fibrous layer of the joint capsule. It contains regularly blood vessels, nerve fibers, lymph vessels and might contain some elastic fibers (1).

According to tissue composition and shape, three distinct types of subintimal stroma are defined: areolar, fibrous and adipose types. The areolar type is composed of loose connective tissue and shows extensive vasculature. It is further characterized by the formation of villous protrusions. The fibrous type shows dense, fibrous connective tissue and poor vascular supply. The subintima of the adipose type consists mainly of fat cells (6). There are also mixed forms of various characteristic types, such as the fibroadipose type.

In humans, the synovial folds are located in the ventral and dorsal aspect of the right and left lateral atlantoaxial joints, respectively (7, 8). These crescent-shaped intra-articular projections originate from the synovial layer and their first description is attributed to Henle in 1855 (3, 9). The folds are subdivided into 3 parts based on their appearance: the base lies directly on the fibrous layer of the capsule, seamlessly joins the middle part, and tapers to an apex that extends into the atlantoaxial joint (Figure 1) (10). Synovial fold volume varies with size and gender, being larger in males (8, 11, 12). While the children population shows a higher number of larger folds than adults, there is no correlation between age and synovial fold volume in adults (2, 11). Ventral synovial folds have been described in human literature to be significantly larger than dorsal folds (11, 12). Adipose synovial folds are commonly found in children and also in the atlantooccipital joint of adults, whereas fibrous or fibroadipose synovial folds are typically observed in the atlantoaxial joint (2, 3). The nerve fibers within the subintimal stroma of the folds are found both around blood vessels and as free fibers, highlighting their roles in vasoregulation and nociception (13), with the ventral rami of the C1 and C2 spinal nerve innervating the atlantoaxial joint capsule (14).

The function of synovial folds is still unclear. They are thought to be “passive space fillers” that fill the noncongruent aspect of the joint and adapt to joint motion and are therefore often called “meniscoids” (3, 7). They therefore act as a protective shield and lubricant for the articular surfaces (3, 7, 9, 15). In humans, several hypotheses have been proposed regarding the potential involvement of synovial folds in disease processes and their clinical relevance. These include synovial fold entrapment and extrapment. Synovial fold entrapment involves the fold being caught between the articular surfaces (10, 16), while extrapment refers to the fold being trapped outside the articulation, between the joint capsule and the vertebra, due to abnormal movement (17, 18). Both conditions are believed to contribute to the development of torticollis (2, 19–21). Additionally, joint immobility has been hypothesized to lead to fibrosis of the synovial folds, which could reduce the range of motion (18). However, the majority of studies on atlantoaxial synovial folds in humans have focused on their role in whiplash injuries (1, 22–26). Autopsy studies have reported contusions or tears of the synovial folds in whiplash patients, which may contribute to cervical pain (22–26) and balance and visual disturbances have been reported to accompany upper cervical pain and are of particular clinical importance in the context of whiplash (27–29).

Given the potential association of synovial fold injury with neck pain in humans, there is growing interest in *in vivo* imaging techniques. Synovial folds were first successfully visualized *in vivo* on MRI using an isotropic three-dimensional T1-weighted spoiled gradient echo volumetric interpolated breath-hold examination fat-suppressed (VIBE) sequence and a T2-weighted sampling perfection with amplification-optimized contrast using different angle evolutions (SPACE) sequence (30). Recent work has attempted to correlate the histological results and MRI findings of synovial folds. Based on signal intensities on VIBE and SPACE sequences, synovial folds could be classified as containing mostly adipose tissue (hyperintense on VIBE and SPACE), mostly connective tissue (hypointense on VIBE and SPACE), or mixed tissue (mixed hyper- and hypointense on VIBE and SPACE sequences), showing a good correlation with histological findings (31). Another method using in-phase, out-of-phase, fat-only, and water-only Dixon MRI images improves agreement with histological findings to a “substantial” level (18, 32).

Despite the wealth of information in human anatomy, the existence, morphology and potential function of synovial folds within the normal canine atlantoaxial joint remains unexplored, with only one mention of synovial protrusions within the atlantoaxial joint in the veterinary anatomy literature (33). Our aim was to provide the first comparative description of synovial folds in the presumably normal canine atlantoaxial joint using advanced MRI imaging techniques, gross anatomical examination, and histological analysis.

## 2 Materials and methods

### 2.1 Case selection and inclusion criteria

This prospective cadaveric study involved dogs euthanized at the Small Animal Teaching Hospital of the Vetsuisse Faculty, University of Bern, Switzerland, with owner consent for research purposes. A total of six dogs were evaluated in this study, including two mixed-breed dogs (dog 2 and 3), one Havanese (dog 6), one Shar Pei (dog 1), one Belgian Shepherd (dog 4), and one Giant Schnauzer (dog 5). Three of the dogs were spayed females, two were neutered males, and one was an intact male. The ages of the dogs ranged from 3.5 months to 13 years, with a median age of 7.5 years. The weight of the dogs ranged from 3 to 34 kg, with a median weight of 23 kg. Although dogs of various sizes, breeds, and ages were considered, practical constraints led to the inclusion of at least one small-breed dog for histological purposes. Dogs were excluded if they had a history of craniocervical disease or trauma. Comprehensive information including breed, age, gender, body weight, and reason for euthanasia was documented (Table 1).

**Table 1:** Summary of comprehensive dog information including breed, age, gender, weight, and diagnosis leading to euthanasia (m = male, mc = castrated male, f = female and fc = spayed females).

Dog	Breed	Age in years	Gender	Weight in kg	Diagnosis
1	Shar Pei	4.4	mc	28.8	Renal insufficiency, suspicion of amyloidosis
2	Mixed-breed	13.0	fc	17.2	Likely neoplastic hepatic mass, possible splenic infiltration, hemorrhagic abdominal effusion.
3	Mixed-breed	6.5	fc	33.0	Disseminated intravascular coagulopathy, cerebral hemorrhage
4	Belgian Shepherd	0.3	m	8.8	Tubulointerstitial nephritis
5	Giant Schnauzer	8.4	m	33.7	Suspicion of primary neoplastic disease in the left caudal lung lobe
6	Havanese	11.9	fc	3.0	Cardiogenic pulmonary edema

All dogs underwent cervical MRI followed by dissection on the same day. Histological evaluation was performed specifically on the single small breed dog. All but one dog were cooled at +6 °C after euthanasia, one dog was frozen at −20 °C for 3 days, and cooled for 1 week prior to MRI and dissection. For histological examination, the dog was cooled at +6 °C underwent MRI 12 h after euthanasia and dissection was performed 15 h after death. The prepared sample was directly fixed in formalin 4%.

### 2.2 Diagnostic imaging

Dogs were positioned in dorsal recumbency for imaging using a 3 T MRI unit (Magnetom Vida, Siemens Healthineers, Erlangen, Germany). The selection of MRI sequences was based on standard protocols for imaging the cervical spine used in our clinic—namely T1-weighted, T1-weighted 3D, T2-weighted, STIR, and Dixon sequences—as well as on previous literature indicating that synovial folds were first successfully visualized *in vivo* using VIBE and SPACE sequences. Accordingly, the study protocol included sagittal and transverse T1-weighted and T2-weighted sequences, a water-only Dixon sagittal sequence, dorsal STIR, and a VIBE sequence. Additionally, a SPACE sequence and a T1-weighted gradient echo 3D MPR were performed in the first two dogs. However, due to low resolution and time constraints, those sequences were subsequently excluded from the standard protocol.

The MRI sequence parameters were as follow:

VIBE fat-saturated: TR/TE 18/3.75 ms, flip angle 9°, field of view (FOV) 179 × 179 mm, matrix 126 × 192, 58 slices (0.7 mm), voxel size 0.6 × 0.6 × 0.7 mm (0.252 mm^3^), acquisition time 4:39 min.
T1w transverse: TR/TE 2950/9.6 ms, FOV 100 mm, matrix 112 × 160, slice thickness 2.5 mm, acquisition time 2:46 min.
T1w sagittal: TR/TE 1800/9.7 ms, FOV 120 mm, matrix 416 × 291, slice thickness 1.5 mm, acquisition time 2:02 min.
T2w transverse: TR/TE 4000/95 ms, FOV 100 mm, matrix 128 × 160, slice thickness 2.5 mm, acquisition time 1:41 min.
T2w sagittal: TR/TE 2800/85 ms, FOV 220 mm, matrix 269 × 384, slice thickness 2.5 mm, acquisition time 1:38 min.
STIR dorsal: TR/TE 4000/37 ms, FOV 240 mm, matrix 211 × 304, slice thickness 3 mm, acquisition time 2:00 min.
DIXON sagittal (“Myelo Sequence”): TR/TE 4010/204 ms, FOV 220 mm, matrix 224 × 238, slice thickness 2 mm, acquisition time 1:58 min.
T1 3D: TR/TE 2400/3.46 ms, flip angle 8°, FOV 125 × 125 mm, matrix 192, 144 slices (0.6 mm), voxel size 0.3 × 0.3 × 0.6 mm (0.054 mm^3^), acquisition time 5:19 min.
SPACE: TR/TE 1300/134 ms, flip angle 100°, FOV 78 × 120 mm, matrix 256 × 256, 46 slices (0.7 mm), voxel size 0.3 × 0.3 × 0.7 mm (0.063 mm^3^), acquisition time 6:38 min.

The FOV and individual parameters of the MRI sequences were adjusted to dog size, resulting in mild variations of the above-mentioned values.

The total acquisition time was 27 min 41 s in the first two dogs, and 15 min 44 s for the other dog.

### 2.3 Anatomical dissection

The cervical spine was either removed en bloc between the external occipital protuberance and the caudal cervical vertebrae, or the head was left attached to the cranial cervical vertebrae, and the resection was performed between the caudal cervical vertebrae. Cutaneous, subcutaneous tissues, superficial muscles, cervical viscera, and blood vessels were meticulously removed along with the cervical musculature down to the Musculus obliquus capitis caudalis dorsally and Musculus longus colli ventrally. After careful dissection of these muscles from the atlantoaxial joint capsule, the capsule was cautiously opened caudally to examine for possible synovial folds.

### 2.4 Histology

The cervical spine was removed en bloc between the external occipital protuberance and the caudal cervical vertebrae. Anatomical preparation was performed as described in point 2.3. The specimen was then fixed in 4% formalin and later decalcified and fixed with Ossa Fixona® (Diagonal GmBH & Co. KG, Münster, Germany) at room temperature. Adequate decalcification status was judged by palpation (soft to elastic bone). A sagittal cut was then made to separate the left and right lateral aspects of the joint. One additional parasagittal cut was performed on the left, and two parasagittal cuts were performed on the right, resulting in two blocks on the left and three blocks on the right side. The blocks were fixed in Formalin 4% for 48 h at 4 °C, put in a cassette, dehydrated in alcohol and then were embedded in Paraffin.

Starting from the parasagittal paraffin block surfaces, further parasagittal sections with a thickness of 2 μm were serially sectioned through the entire atlantoaxial joint to demonstrate the structure and evolution of the folds on a histological level.

The sections were then mounted on slides, stained with hematoxylin and eosin (HE) or with Masson-Goldner-Trichrome and captured using a digital microscope (Axio Imager, Carl Zeiss Vision Swiss AG, Feldbach, CH).

## 3 Results

### 3.1 Diagnostic imaging

**Figure 2:**
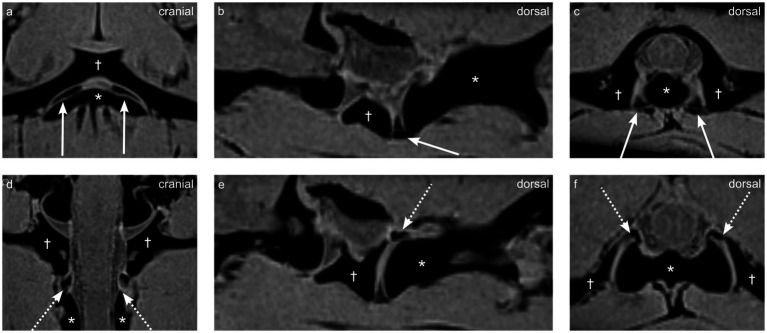
Multiplanar reconstructions from a VIBE sequence demonstrating synovial fold anatomy in dog 1 (4 years old Shar Pei), the atlas is indicated by the dagger (†), and the axis by the asterisk (*). **(a,d)** Dorsal views, **(b,e)** parasagittal views, and **(c,f)** transverse views highlight the ventral synovial folds (arrows **a–c**) and dorsal synovial folds (dashed arrows **d–f**).

Ventral synovial folds were consistently visible on fat-suppressed VIBE sequence and appeared as sharply delineated, triangular structures emerging ventrally from the joint capsule on both the right and left sides and located between the articular surfaces of the atlantoaxial joint as seen on Figures 2a–c.

They appeared homogeneously strongly hypointense on VIBE fat-suppressed sequence and contrasted sharply with the surrounding hyper-to isointense synovial fluid (Figure 2).

**Figure 3:**
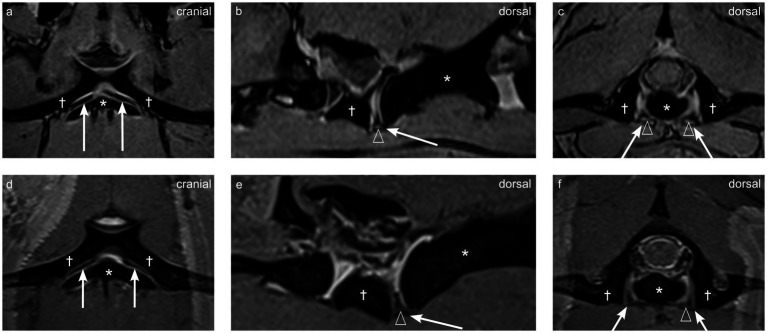
Multiplanar reconstructions of synovial folds using T1-weighted and SPACE sequences in dog 1 (4 years old Shar Pei), the atlas is indicated by the dagger (†), and the axis by the asterisk (*). **(a-c)** T1-weighted sequence showing dorsal **(a)**, parasagittal **(b)**, and transverse **(c)** views. **(d-f)** SPACE sequence showing corresponding dorsal **(d)**, parasagittal **(e)**, and transverse **(f)** views. Ventral synovial folds indicated by arrows, and central hyperintensity indicated by empty arrowheads.

In the two dogs (dogs 1 and 2) where SPACE and a 3D T1 MPR sequence were available, the synovial folds were peripherally hypointense, and centrally hyperintense compared to the paravertebral musculature as visible on Figure 3. The synovial folds consistently showed T2w, Dixon water only hypointensity, T1w hypo- to isointensity, and STIR marked hypointensity relative to the musculature, but were not clearly delineated on these sequences as visible on Figure 3.

Dorsal synovial folds were clearly visible in three dogs (dogs 1, 4 and 5) and were triangular in shape, arising from the dorsal right and dorsal left lateral aspects of the joint capsule and slightly protruding between the articular surfaces and are shown in Figures 2d–f. Although smaller than the ventral folds, they were of similar intensity. In the remaining dogs, some strongly hypointense, ill-defined small structures were also seen at the same location. A summary of the magnet resonance imaging findings is provided in Table 2.

**Table 2:** Summary of magnetic resonance imaging sequences and visualization of atlantoaxial synovial folds in each dog.

Dog	1	2	3	4	5	6
Visibility of ventral synovial folds	Consistently visible on fs VIBE sequence, appeared as sharply delineated, triangular structures emerging ventrally from the joint capsule between the articular surfaces of the atlantoaxial joint					
Visibility of dorsal synovial folds	Clearly visible, as ventral, emerging dorsally from the joint capsule	Not visible	Ill-defined	Clearly visible, as ventral, emerging dorsally from the joint capsule	Clearly visible, as ventral, emerging dorsally from the joint capsule	Not visible
SI in fs VIBE sequence	Homogeneously strongly hypointense					
SI in T1w sequence	Peripherally hypointense and centrally hyperintense. Well delineated on 3D sequence, not clearly delineable on T1w transverse images		Not clearly delineable, hypo- to isointense			
SI in T2w sequence	Not clearly delineable, mildly hypointense					
SI in Dixon sequence	Not clearly delineable, hypointense					
SI in SPACE sequence	Peripherally hypointense and centrally hyperintense		Not available			
SI in STIR sequence	Not clearly delineable, hypointense					

The signal intensity is compared to the paravertebral musculature (SI = signal intensity, fs = fat-suppressed).

### 3.2 Anatomical dissection

**Figure 4:**
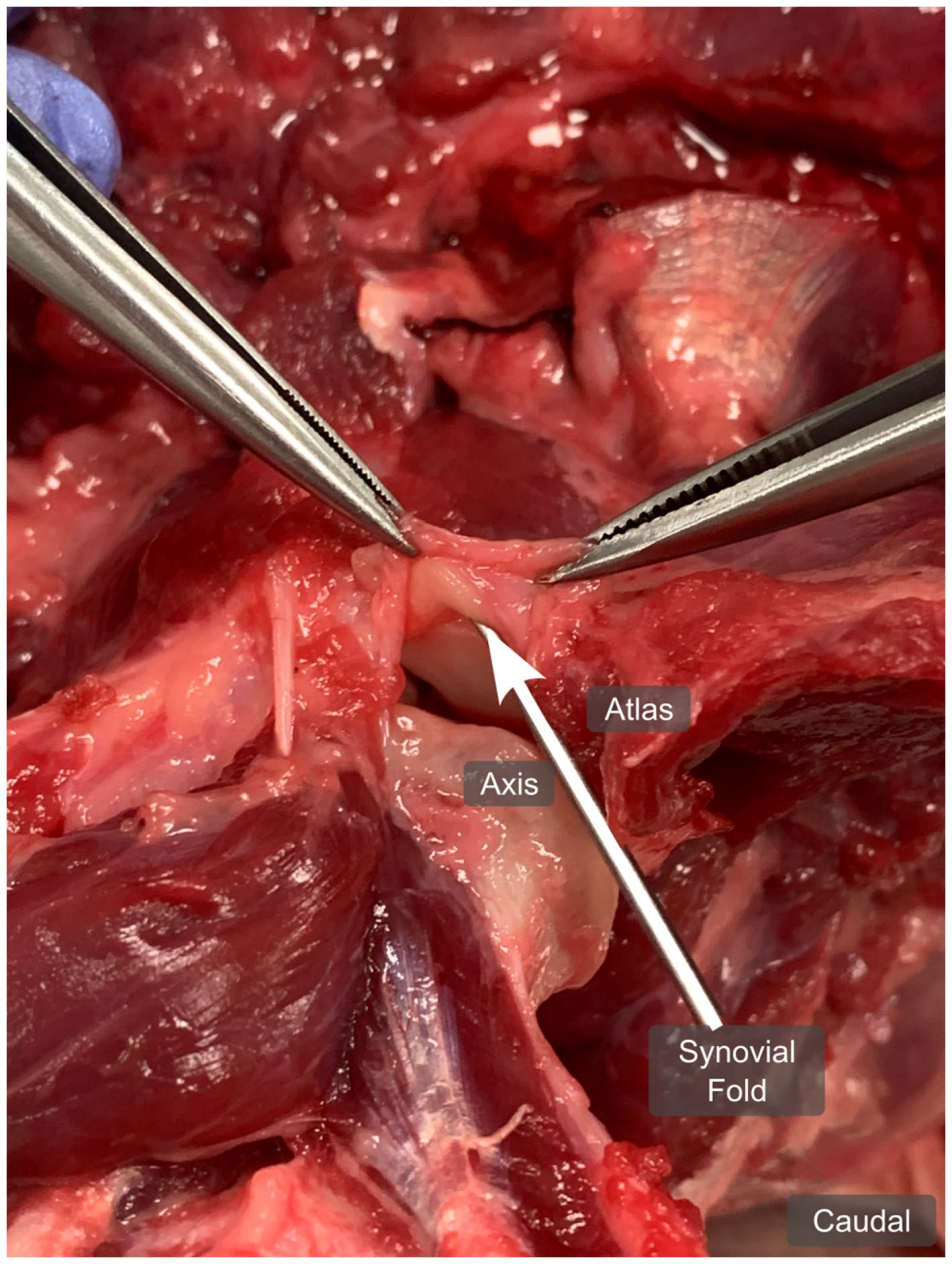
Anatomical dissection of the left atlantoaxial joint from dog 1 (4 years old Shar Pei), showing the left ventral synovial fold (arrow) from ventrally, in a dorsal plane. The joint capsule is open and pulled cranially.

During anatomical dissection, a broad-based, crescent-shaped, fat-like structure originating from the fibrous atlantoaxial joint capsule was observed in the ventral and dorsal aspects of the left and right lateral atlantoaxial joints, respectively as seen in Figure 4. Upon opening the joints, these structures were situated between the articular surfaces. When manipulated, by pulling and restoring them to their normal position (closed joint), these structures reinserted between the articular surfaces, leading us to identify them as synovial folds. The dorsal synovial folds were consistently smaller in size than their ventral counterparts, often only recognizable as focal thickening and slight bulging of the fibrous joint capsule within the joint space.

### 3.3 Histology

The microscopic examination revealed ventral and dorsal synovial folds with various dimensions. Larger folds, which protruded deep into the intraarticular space and smaller ones, which build only elevations, were observed. The larger folds confirmed regularly the subdivision into three parts; the base, the middle and the apex. Interestingly, subintimal stromal content of all folds varied from level to level in the course of the joint space and could be assigned mainly to one of the three characteristic types: the fibrous, the areolar and the adipose type; mixed forms were also observed.

The following description of the morphology and tissue composition of the ventral fold refers primarily to the largest of the folds.

**Figure 5:**
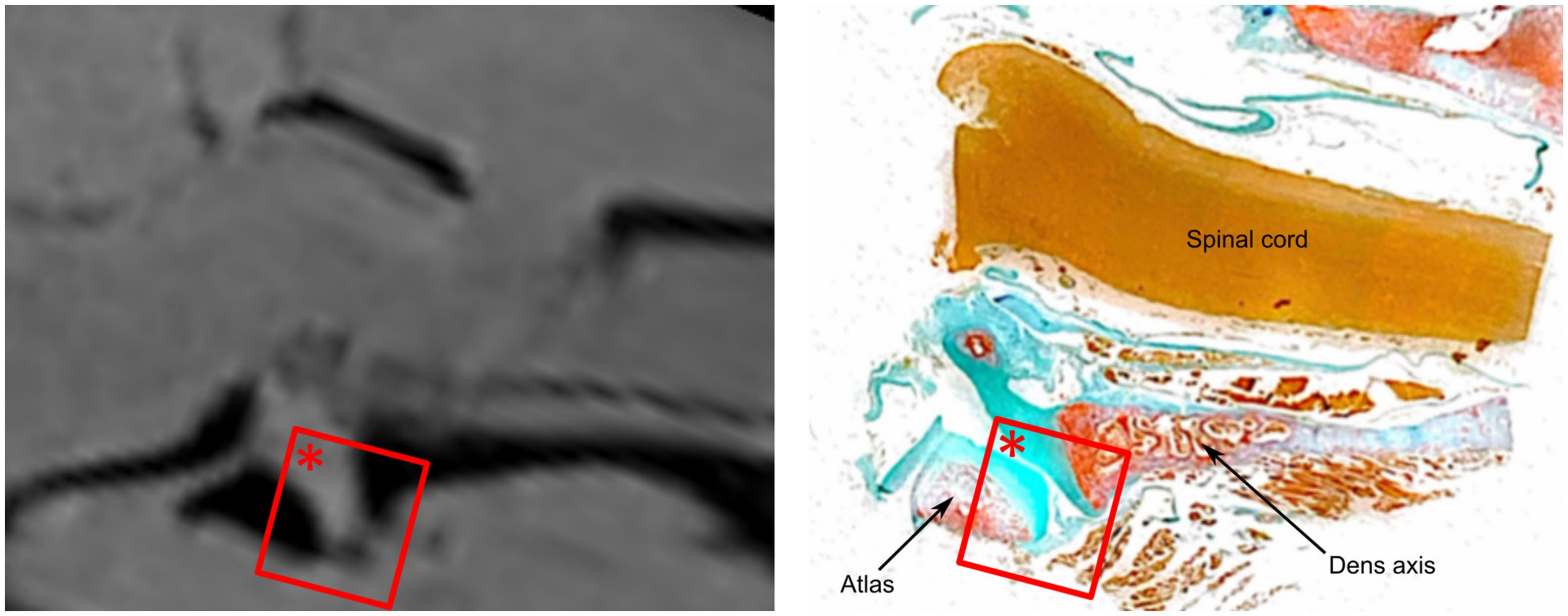
Comparative analysis of the ventral synovial fold in dog 6 (12 years old Havanese). Parasagittal MRI VIBE sequence (left) is juxtaposed with the corresponding parasagittal histological section (right, LM, Goldner stain, 10x).

**Figure 6:**
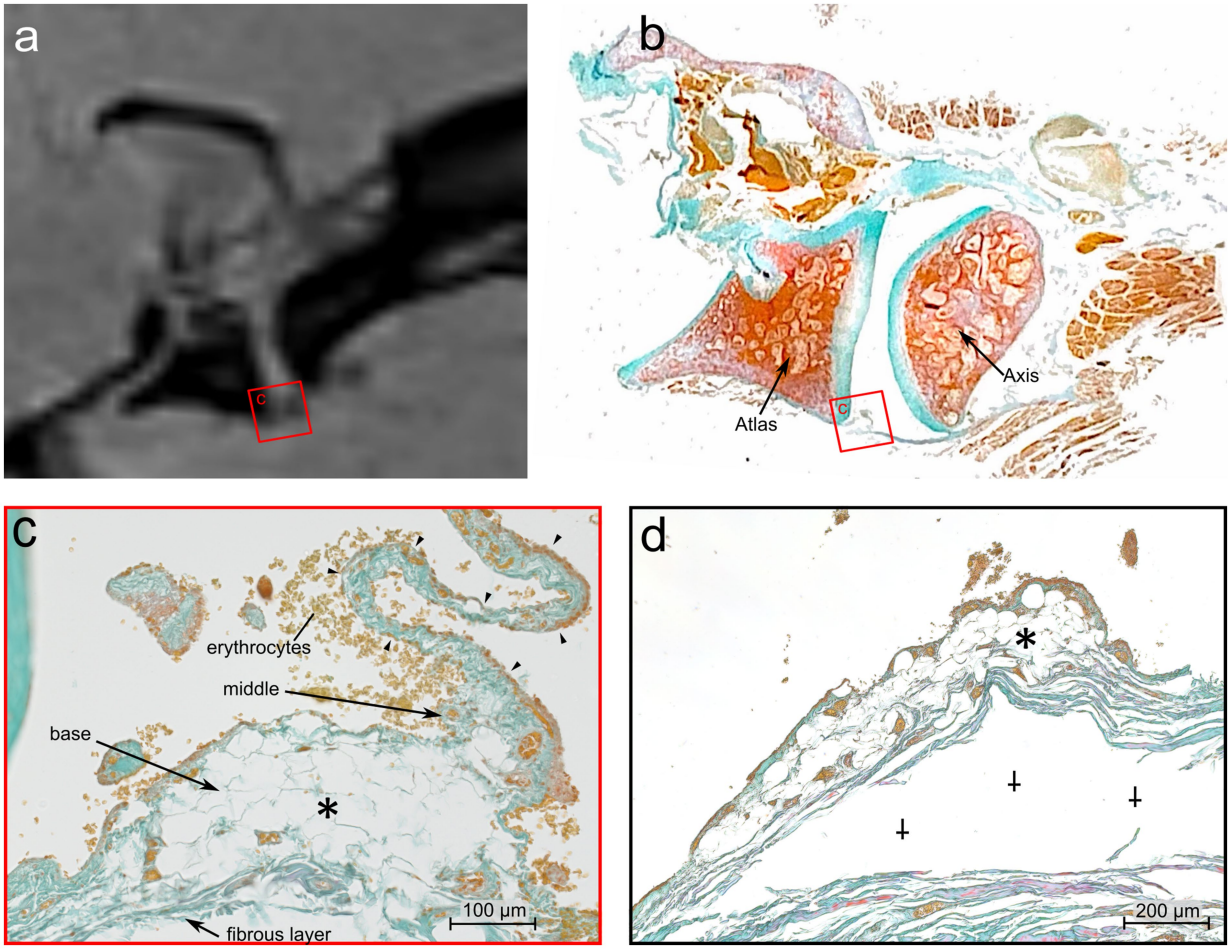
Comparative MRI and histologic analysis of the ventral synovial fold in the atlantoaxial joint of Dog 6, a 12 years old Havanese. **(a)** Parasagittal MRI VIBE sequence juxtaposed with **(b)** at the mid-level of the lateral part of the atlantoaxial joint showing the ventral synovial fold (square) (LM, Goldner stain). **(c)** The ventral synovial fold is shorter with a broad base, and an apex which is indicated by arrowheads. The fold is composed of adipose tissue at its base (*) (LM, Goldner stain, 40x). **(d)** Further laterally, the apex of the fold is no longer visible, and the base is mainly composed of adipose tissue (*). Artifactual detachment of the fibrous capsule is noted (⸸) (LM, Goldner stain, 40x).

A continuous large ventral fold was identified at four different longitudinal sections through the atlantoaxial joint, starting at the level of the dens axis and continuing laterally from there towards the level of the transverse foramen of the axis, which corresponds approximately to the lateral end of the atlantoaxial joint cavity. The location and shape of this ventral synovial fold corresponded to the structure seen in the MRI (Figures 5, 6a).

**Figure 7:**
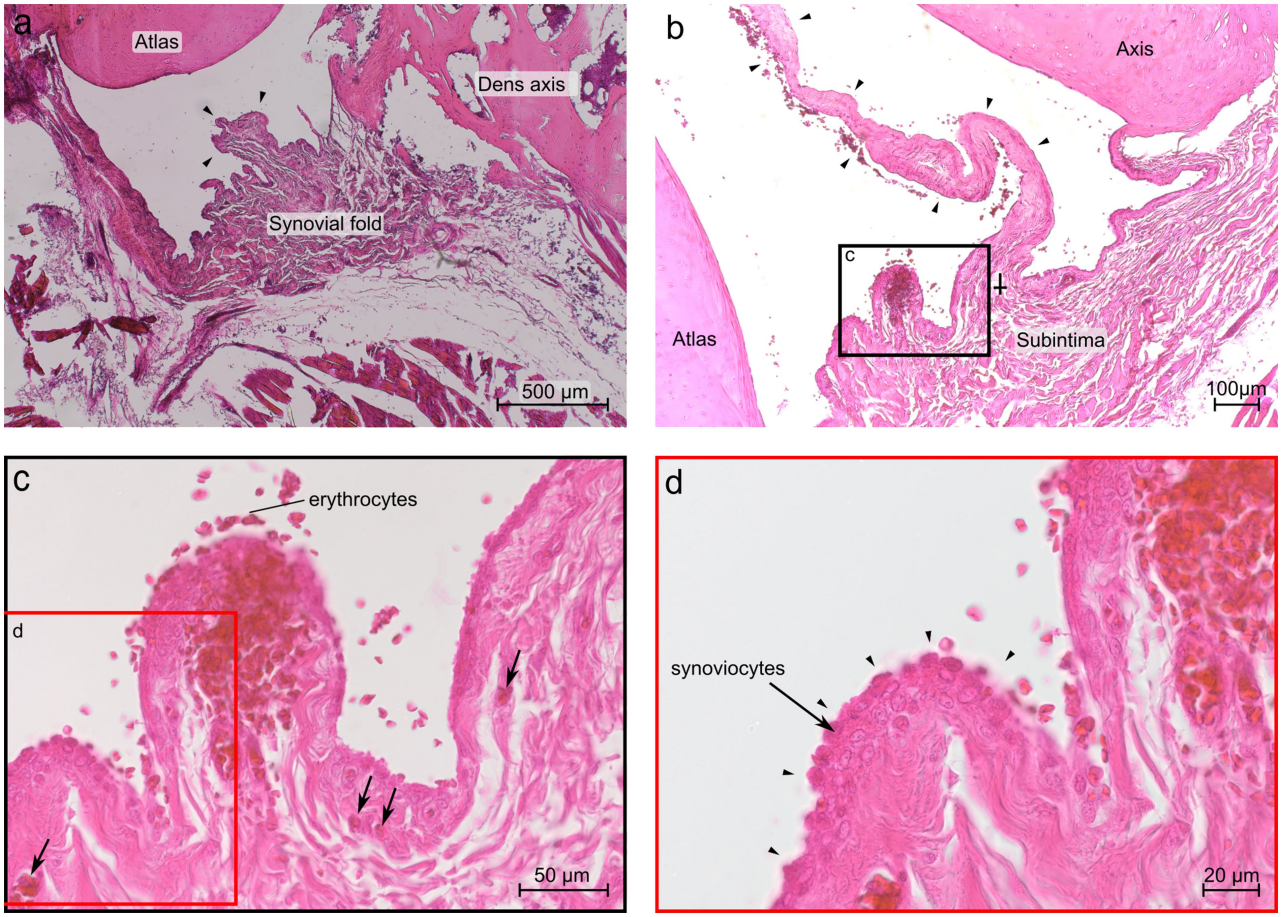
Histological section of the ventral synovial fold in the atlantoaxial joint of dog 6 (12 years old Havanese). **(a)** Sagittal section at the level of the dens axis: the synovial fold shows a thick base and a substantial middle portion, comprising strands of connective tissue that extend toward the apex (arrowheads) (LM, H&E stain, 40x). **(b)** Parasagittal section just lateral to the dens axis, the fold is thinner and elongated (arrowheads), and the base exhibits a distinct subintimal layer, diminishing in prominence toward the middle portion of the fold (⸸) (LM, H&E stain, 40x). **(c)** Blood vessels are visible within the fold as indicated by the arrows (LM, H&E stain, 40x). **(d)** Higher magnification reveals that up to three cell layers of synoviocytes form the intima, as indicated by arrowheads (LM, H&E stain, 63x).

At the level of the dens axis the subintima of the ventral synovial fold comprised densely packed fibrous strands and poor vasculature (Figure 7a). The synoviocytes built a continuous superficial layer along the entire fold. The subintimal stroma at the base was formed by fibrous strands that were in continuous alignment with the *Stratum fibrosum* of the joint capsule. The *Stratum fibrosum* of the joint capsule was composed of densely packed fibrous strands containing blood vessels. In the middle part of the fold the fibrous strands changed their direction and were oriented towards the apex, which protruded between the articulating parts of the joint. Bilaterally to the large ventral fold were several short synovial protrusions.

The subintima of the large ventral fold in the region laterally to the dens axis showed fibrous strands at the base aligned with the *Stratum fibrosum* of the joint capsule, but were more loosely packed and more vascularized, compared to the fibers of the fold at the level of the dens axis (Figure 7b). The middle part of the fold comprised densely packed, nearly axial arranged fibers. Blood capillaries were mainly located beneath the synovial cells. The apex of the largest protrusion lacked a distinct subintimal layer. Instead, the opposite layers of synovial cells came into close contact so that the intimal layer appeared to be thicker here (Figure 7b).

Bilaterally to the large ventral fold were several shorter protrusions but were fewer in numbers compared to the level at the dens axis. The shorter protrusions contained loose connective tissue and a rich vasculature (Figure 7c). The synovial cells of the shorter protrusions formed up to three cell layers (Figure 7d).

Sections of the ventral fold at a more lateral level of the atlantoaxial joint (Figures 6b,c) showed a base that consisted almost entirely of fat cells, and thus showed a clear demarcation to the *Stratum fibrosum* of the joint capsule. Also, the middle part of the fold was mainly composed of fat cells. Both, the base and the middle part, were well vascularized. The comparable thin, elongated apex protruded towards the intraarticular space. It was interspersed by densely packed, axial oriented fibrous strands and blood capillaries.

Sections of the ventral fold cut in the periphery of the atlantoaxial joint (Figure 6d) consisted of fat cells and blood vessels, but a clear distinction between the three parts of the fold was not possible at this level.

**Figure 8:**
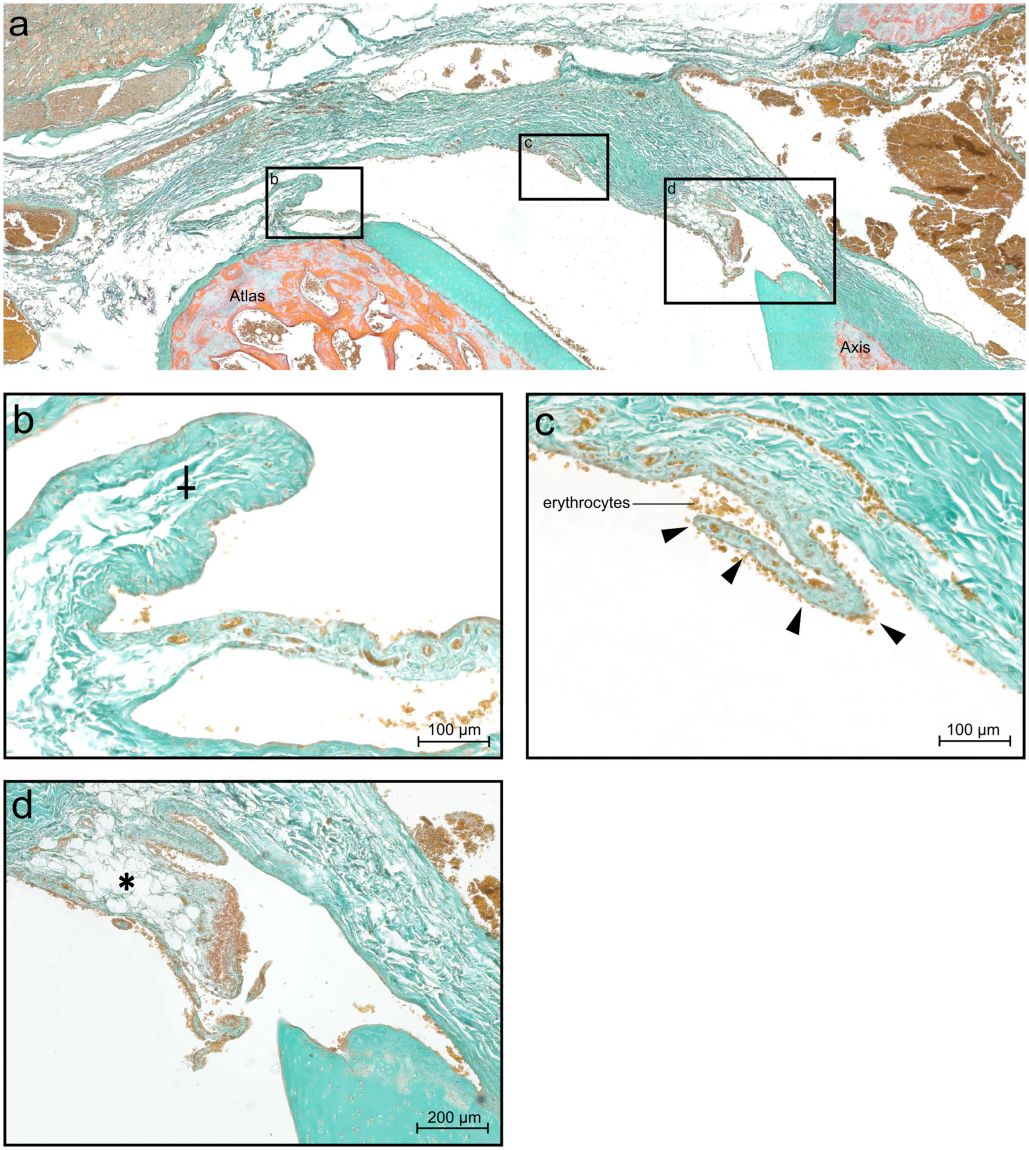
Histological section of the dorsal synovial fold in the atlantoaxial joint of dog 6 (12 years old Havanese). **(a)** Parasagittal histological section stained with Goldner stain, showing the dorsal synovial fold and synovial protrusion (LM, Goldner stain,10x, panorama reconstruction). **(b)** Cranially located synovial protrusion (⸸), not positioned between the articular surfaces (LM, Goldner stain, 20x). **(c)** Arrowheads point at a synovial protrusion at the base of the dorsal synovial fold, showing an intimal layer without a discernible subintimal layer (LM, Goldner stain, 20x). **(d)** Dorsal synovial fold, an abrupt transition at the junction to the middle section is seen, shifting from connective tissue to adipose tissue (*) (LM, Goldner stain, 20x).

In contrast to the large ventral fold, a dorsal fold could not be identified at the level of the dens axis but was present in lateral sections. However, several synovial protrusions with varying size were present dorsally. The *Stratum fibrosum* of the joint capsule was composed of densely packed fibrous strands containing blood vessels. The tissue composition of different shaped protrusions varied. There were short protrusions with a broad base and elongated protrusions, both types comprised loose, well vascularized connective tissue (Figures 8a–c). Also, a comparatively larger protrusion with a base of densely packed fibrous strands, which were mixed up with fat cells and blood vessels was visible in the middle part (Figure 8d).

## 4 Discussion

Our findings demonstrate that synovial fold are consistent anatomical components of the canine atlantoaxial joint, with morphology and tissue composition comparable to those described in humans, and highlights the potential for high-field MRI—particularly fat-suppressed VIBE sequences—to visualize these structures.

Synovial folds of the canine atlantoaxial joint were visible on MRI in dog cadavers without known atlantoaxial disease, with the best visualization achieved using the VIBE sequence. In this sequence, fat and fibrous tissue- the primary components of synovial folds- appear hypointense, whereas the surrounding structures such as synovial fluid are isointense to the musculature, leading to an increased contrast between the synovial folds and the surrounding structures. Furthermore, the VIBE sequence provides high spatial resolution three dimensional images, which facilitates the visibility of small anatomical structures. Additionally, imaging was performed using a 3 Tesla MRI system, which offers high spatial resolution and likely contributed to the clear visualization of these small structures. Although synovial folds are known to be visible on 1.5 Tesla MRI in humans, it remains uncertain whether similar visibility could be achieved in dogs—particularly given their generally smaller size—or when using lower-field MRI systems.

In all dogs examined, ventral synovial folds were identified by MRI and confirmed by anatomical dissection. While dorsal synovial folds were visible on MRI only in three dogs, their presence was confirmed in all dissected dogs.

In all dogs examined, dorsal synovial folds were smaller than the ventral synovial folds, which is also consistent with previous findings in humans (11, 12).

**Figure 9:**
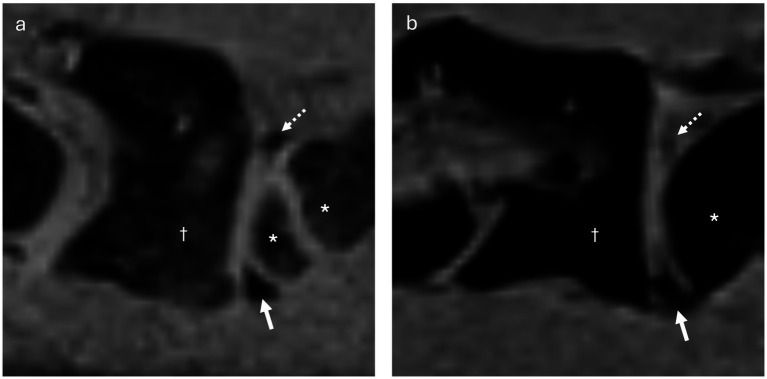
**(a)** Parasagittal VIBE image of the atlantoaxial joint of dog 4 (3 months old Belgian Shepherd, with an open growth plate in the cranial aspect of the axis), showing well-delineable, rather large ventral (arrow) and dorsal (dashed arrow) synovial folds. The atlas is indicated by the dagger (†), and the axis by the asterisk (*). **(b)** Parasagittal VIBE image of the atlantoaxial joint of dog 3 (6 years old Mixed breed dog), showing ill-delineable, smaller ventral (arrow) and dorsal (dashed arrow) synovial folds. The atlas is indicated by the dagger (†), and the axis by the asterisk (*).

The lack of visible dorsal synovial folds on MRI in the remaining 3 dogs was likely attributable to their small size and individual anatomical variation, particularly related to overall body size. This became obvious as dorsal synovial folds were clearly delineated in larger dogs (dog 1, Shar Pei and dog 5, Giant Schnauzer). Notably, dog 5, the largest, had also the highest body weight. However, dog 3, a mixed-breed dog, despite weighing more than dog 1, did not exhibit clearly delineable dorsal synovial folds (Figure 9). This discrepancy might be breed-specific, related to different composition of the folds or attributed to age differences as described in humans, were older individuals tend to show smaller synovial folds (7), similarly dog 1 was 4.4 years old, while dog 3 was 6.5 years old. The youngest dog in this study, a 4-month-old puppy, had large and well-defined dorsal and ventral synovial folds (Figure 9). This observation suggests that, similar to observations in humans, synovial folds may also be larger in young than in adult dogs (2, 11). In some dogs, ill-defined fat-suppressed, VIBE strongly hypointense structures were visible at the expected location of the dorsal synovial folds, suggesting their presence, which was confirmed in anatomical dissection in all but one dog, where it was confirmed on histology.

In humans, the synovial folds are also believed to be more adipose in composition at a young age (2).

The above-described individual factors, combined with the limited spatial resolution of MRI, may have contributed to the inability to consistently visualize these small structures.

One limitation of the present study is that all examinations were performed on cadaveric specimens. Although a high-resolution protocol using a 3 Tesla MRI system was employed, it remains possible that post-mortem changes, including those related to prior freezing and thawing, may have introduced artifacts—such as abnormal positioning or altered signal intensity of the synovial folds. Additionally, the absence of normal perfusion may lead to a signal alteration. Contrast-enhanced imaging with gadolinium, which is routinely used in clinical veterinary MRI, was not performed. As a result, we were unable to evaluate the behavior or enhancement pattern of synovial folds following contrast administration.

To better characterize these structures, this study provides a detailed anatomical description of the shape and architecture of the larger ventral synovial folds, which were consistently visible on MRI.

Complementary histological analysis further elucidated the morphology and tissue composition of the synovial folds at the cellular level.

Notably, the shape of the canine synovial fold, especially the ventral fold permitted subdivision into three parts: (1) base, (2) middle, (3) apex, corresponding to the observation in humans (10). In addition, we were able to assign the three types of subintimal stroma described in humans—areolar, fibrous and adipose—to the canine synovial folds on the basis of the tissue composition (6). The synovial folds in this study were described at different levels of the atlantoaxial joint and, interestingly, the composition of the subintimal stroma varied depending on the location in the joint. At the level of the dens axis the subintima of the ventral synovial fold showed characteristics of the fibrous type with densely packed fibrous strands and poor vasculature. Although the subintima of the large ventral fold in the area lateral to the dens axis had fibrous strands at the base which were more loosely packed and more vascularized than the fibers of the fold at the level of the dens axis, the middle part of the fold in particular consisted of densely packed, almost axially arranged fibers representing the fibrous type. Sections of the ventral fold at a more lateral level and in the periphery of the atlantoaxial joint exhibited features consistent with the adipose type. Studies of synovial folds in humans have also described mixed forms of the various characteristic types, such as the fibroadipose type, and the subintimal stroma also regularly contains blood vessels, nerve fibers, lymphatic vessels and elastic fibers (1). However, it is not yet clear from the literature whether the mixed types are observed in humans at specific localizations or whether—as in our case—the composition of the tissue changes over the course of the synovial fold.

In addition to the presence of dorsal synovial elevations, which could be classified as areolar due to the loose, well-vascularized stroma, a comparatively longer dorsal synovial protrusion was observed. This protrusion had a base consisting of densely packed fibrous strands and was intermixed with fat cells and blood vessels in the middle portion. Consequently, this fold corresponded to a fibroadipose subintimal type.

Altogether, the histological observations of the atlantoaxial joint’s synovial folds in that dog correspond to the findings in humans, where fibrous and fibroadipose synovial folds are typically observed in the atlantoaxial joint (6). The occurrence of different tissue composition and shapes allows for the hypothesis that different mechanical stresses act on the folds. Smaller elevations, protrusions and folds presumably serve to increase the surface area of the synovial layer, which also represents the site of production for synovial fluid.

In human medicine, synovial folds are hypothesized to function analogously to menisci, contributing to joint congruity and stability, protecting and lubricating the articular surface, assisting in weight-bearing, and dissipating mechanical stress (3). Our investigations have demonstrated that the largest synovial folds protrude into the joint space, i.e., between the articulating surfaces. This suggests an important function in maintaining joint congruity and/or enhancing the weight dissipation between the articulating bones.

In addition, we also observed many smaller protrusions of the synovial layer, without reaching into the joint space. Here, their function is probably more to increase the surface area and therefore support lubrication.

Nevertheless, it is not possible to draw definitive conclusions about the general histological structure of the synovial folds from one dog. Further research based on more animals is required to elucidate the differences in size and composition of synovial folds between age groups and breeds in dogs.

In humans, the clinical relevance of synovial folds stems from the presence of nociceptive nerve fibers. Additionally, there is ongoing debate regarding whether synovial folds contribute to proprioceptive feedback for sensorimotor control, potentially establishing neurophysiological connections to the vestibular and visual systems via cervical proprioceptors (34, 35). However, with the methods we used on the one specimen, we could not clearly identify nerve fibers. Further investigations including specific histological staining or immunohistochemistry are required.

Beyond histology, the tissue composition of synovial folds, as evaluated via MRI, has attracted growing clinical interest—particularly in human medicine. Current research is investigating correlations between MRI signal characteristics of synovial folds on specific sequences (e.g., Dixon, VIBE, and SPACE) and histological findings (18, 31, 32). In our study, the ventral folds of dog 1 exhibited homogeneous strongly hypointense signal on the VIBE sequence by MRI imaging, while the central portion appeared partially hyperintense on both T1w and SPACE sequences. This imaging pattern suggested the presence of central adipose tissue. Further, the peripheral regions of the ventral folds were hypointense on T1w and SPACE sequences in dog 1, which is indicative of fibrous tissue. These MRI imaging findings were consistent with the histological results from dog 6, where the synovial folds were found to have a fibroadipose composition. This concurrence suggests that it may be possible to determine the composition of the synovial folds in dogs based on its MRI signal in different sequences as described in humans (18, 31, 32).

The potential clinical utility of such imaging assessments is highlighted by a recent MRI study comparing synovial folds in whiplash-injured people with those in an age-matched control group, showing that the lateral atlantoaxial synovial folds in the whiplash group had an increased fibrous component and were shorter, which suggests a composition alteration and could be of clinical significance in cases of chronic whiplash-associated disorders (1, 8).

Although whiplash injuries are less common in dogs, the anatomical structure of the synovial folds indicates a similarity to humans, suggesting a similar function, and that certain diseases or mechanical impacts can affect the synovial folds of the dog in a similar way or that similar adaptation mechanisms can occur. For example, small breed dogs often suffer from atlantoaxial instability (36), which could lead to increased pressure on the synovial folds due to increased movement or abnormal positioning. Furthermore, the use of collars in dogs may exert additional pressure on these structures, potentially leading to chronic changes.

## 5 Conclusion

Our study offers novel insights into veterinary medicine by being the first to document the presence of synovial folds in the atlantoaxial joint of dogs on 3 T MRI, being best visible on VIBE sequence. We provide detailed information on their localization, size and tissue composition. Thus, this study contributes to a better understanding of canine synovial folds. Future research is needed to investigate the morphology and MRI characteristics of atlantoaxial synovial folds in dogs of different ages, different clinical histories and different breeds to shed light on their anatomical variability and possible involvement in clinically significant disorders.

## Data Availability

The raw data supporting the conclusions of this article will be made available by the authors, without undue reservation.
